# Association between the GRM7 rs3792452 polymorphism and attention deficit hyperacitiveity disorder in a Korean sample

**DOI:** 10.1186/1744-9081-9-1

**Published:** 2013-01-07

**Authors:** Subin Park, Sun-Woo Jung, Boong-Nyun Kim, Soo-Churl Cho, Min-Sup Shin, Jae-Won Kim, Hee Jeong Yoo, Dae-Yeon Cho, Un-Sun Chung, Jung-Woo Son, Hyo-Won Kim

**Affiliations:** 1 Department of Child and Adolescent Psychiatry, Seoul National University Hospital, Seoul, Korea; 2 Department of Psychiatry, Seoul National University Bundang Hospital, Seongnam, Gyeonggi, Korea; 3 Lab Genomics Clinical Research Institute, Seoul, Korea; 4 Department of Psychiatry, Kyungpook National University Hospital, Daegu, Korea; 5 Department of Psychiatry, Chungbuk National University Hospital, Cheongju, Korea; 6 Department of Psychiatry, Ulsan University College of Medicine, Asan Medical Center, Seoul, Korea

**Keywords:** Attention-deficit/hyperactivity disorder (ADHD), Transmission disequilibrium test, Continuous performance test, Genetic polymorphism, Anxiety

## Abstract

**Background:**

The purpose of this study was to investigate the association between the ionotropic and glutamate receptors, N-methyl D-asparate 2A (GRIN2A) and 2B (GRIN2B), and the metabotropic glutamate receptor mGluR7 (GRM7) gene polymorphisms and attention-deficit hyperactivity disorder (ADHD) in Korean population.

**Methods:**

We conducted a case–control analysis of 202 ADHD subjects and 159 controls, performed a transmission disequilibrium test (TDT) on 149 trios, and compared scores from the continuous performance test (CPT), the Children’s Depression Inventory (CDI), and the State-Trait Anxiety Inventory for Children (STAIC) according to the genotype of the glutamate receptor genes.

**Results:**

There were no significant differences in the genotype or allele frequencies of the GRIN2A rs8049651, GRIN2B rs2284411, or GRM7 rs37952452 polymorphisms between the ADHD and control groups. For 148 ADHD trios, the TDT analysis also showed no preferential transmission of the GRIN2A rs8049651 or GRIN2B rs2284411 polymorphisms. However, the TDT analysis of the GRM7 rs3792452 polymorphism showed biased transmission of the G allele (χ2 = 4.67, p = 0.031). In the ADHD probands, the subjects with GG genotype in the GRM7 rs37952452 polymorphism had higher mean T-scores for omission errors on the CPT than did those with the GA or AA genotype (t = 3.38, p = 0.001). In addition, the ADHD subjects who were homozygous for the G allele in the GRM7 rs37952452 polymorphism had higher STAIC-T (t = 5.52, p < 0.001) and STAIC-S (t = 2.74, p = 0.007) scores than did those with the GA or AA genotype.

**Conclusions:**

These results provide preliminary evidence of an association between the GRM7 rs37952452 polymorphism and selective attention deficit and anxiety found within the Korean ADHD population.

## Background

Attention-deficit hyperactivity disorder (ADHD) is a disorder primarily characterized by inattention, impulsivity, and hyperactivity and has a worldwide prevalence of 5.3% [1]. It has an estimated heritability of approximately 76% and is thought to be a complex, polygenic disorder [2].

Although the exact etiology of ADHD is unknown, one theory posits that the dysregulation of neurotransmitter systems underlies the pathogenesis and associated cognitive and locomotor deficits of this disorder [1]. Previous pharmacological and animal knockout studies have strongly implicated dopaminergic and noradrenergic systems in ADHD [3-5]. In addition, similar types of studies support involvement of the glutamatergic system in behavioral models related to ADHD. Magnetic resonance spectroscopy showed an increased glutamatergic tone in the frontal and striatal brains of subjects with ADHD [6-8] that normalized with stimulants and atmoxetine [9]. Dysregulated expression of glutamatergic pathway genes has been observed in spontaneously hypertensive rat models [10,11]. Increased concentrations of glutamate were also reported in the neurometabolism of ADHD brains, which is consistent with altered glutamate transmission in ADHD [8].

Glutamate is the primary excitatory neurotransmitter in the brain and is found as a neurotransmitter in approximately 60% of brain neurons, including almost all cortical pyramidal neurons. Glutamate receptors are responsible for the majority of excitatory synaptic transmissions and plasticity in the central nervous system [12]. Because of this central role in neuronal communication and synaptogenesis, glutamate receptors control several cellular and cognitive processes [13]. Glutamate mediates its effects on the central nervous system via both ionotropic and metabotropic receptors. Ionotropic glutamate receptors are subdivided into three categories based on their respective agonists, namely, a-amino-3-hydroxy-5-methyl-4-isoxazole propionic acid, kainate or N-methyl-D -aspartate (NMDA). The classic learning and memory receptors, NMDA receptors (NMDARs), are composed of heteromeric complexes containing an obligatory NR1 (GRIN1) subunit, plus an additional NR2 (GRIN2A-D) or NR3 (GRIN3A, B) subunit (Riedel et al. 2003). The metabotropic glutamate receptors (mGluRs), which are G-protein-coupled receptors, are divided into 3 groups on the basis of sequence homology, putative signal transduction mechanisms, and pharmacologic properties [14,15]. The mGluRs in group I are mGluRs 1 and 5; mGluRs 2 and 3 in group II; and mGluRs 4, 6, 7, and 8 in group III. Group II and group III mGluRs are linked to inhibition of the cyclic AMP cascade but differ in their agonist selectivities. Among these mGluRs, mGluR7 is the most highly conserved mGluR subtype across mammalian species [16].

The NMDAR subunits GRIN2A and GRIN2B play essential roles in memory and learning by regulating key aspects of synaptic plasticity (Kutsuwada et al. 1996; Tang et al. 1999). Mice lacking the NMDAR 2A subunit gene GluRε1 (the mouse homolog of the GRIN2A gene in humans) show increased spontaneous locomotor activity in novel environments, impaired latent learning associated with selective attention [17] and deficits in spatial learning [18]. Overexpression of the NMDAR 2B subunit gene GluRε2 (the mouse homolog of the GRIN2B gene in humans) in the forebrain of mice enhanced hippocampal long-term potentiation, spatial learning and memory and improved learning processes involved in fear extinction [19]. Knockout studies of GluRε2 indicate that this subunit is essential for development, neuronal patterning and synaptic transmission in the CA1 region of hippocampal slices [20]. The GRIN2A exon 5 polymorphism was associated with ADHD in two of three family studies [21-23], and GRIN2B SNPs showed significantly biased transmission, with the strongest evidence of association found for rs2284411 in ADHD families [24].

It is of particular interest that the metabotropic glutamate receptor mGluR7 (GRM7) gene is widely expressed in the cerebral cortex, hippocampus, and cerebellum, and studies have established a relationship between structural differences in these areas and ADHD [25,26]. Furthermore, mGluR7 has putative roles in anxiety, fear responses, and working memory [27-30]. Deficits in working memory have been identified as potential core factors in the development of ADHD, and anxiety is also a frequently associated condition. Although relational studies investigating GRM genes that encode mGluR have reported mixed results, a Genome-Wide Association Study examining the methylphenidate response in children with ADHD found an association with an SNP in GRM7 gene (rs3792452) [31].

The above-mentioned evidence suggests that GRIN2A, GRIN2B, and GRM7 represent candidate genes for ADHD or certain phenotypes of ADHD. However, the few association studies investigating glutamate genes have yielded mixed results, and all of the above-mentioned studies were conducted on Caucasian populations. Therefore, the purpose of this study was to examine the relationship between ADHD and three glutamate genes: SNPs-GRIN2A exon 5 gene SNP (rs8049651), GRIN2B gene SNP (rs2284411), and GRM7 gene SNP (rs3792452). We conducted a case–control and family-based association study and compared the results of a continuous performance test and clinical scale according to each genotype. We selected these three glutamate genes because of their historical prevalence in the literature examining glutamate genes in relation to ADHD.

## Methods

### Subjects

We recruited children with ADHD from three child psychiatric clinics at university hospitals in Korea: Seoul National, Kyungpook National, and Chungbuk National Hospitals. Inclusion criteria were (1) diagnosis of ADHD according to the Diagnostic and Statistical Manual of Mental Disorders, 4th edition, text revision (DSM-IV-TR) [32]; (2) a score above the 90th percentile on the ADHD Rating Scale-IV (ARS) [33]; (3) a T-score greater than 60 on the Attention Problems subscale of the Child Behavior Checklist (CBCL) [34]; and (4) an intelligent quotient (IQ) higher than 71 on the Korean Educational Developmental Institute's Wechsler Intelligence Scale for Children (KEDI-WISC) [35].

In addition, we recruited control group subjects from five different administrative regions in Korea: Seoul, Seongnam, Incheon, Ulsan, and Yeoncheon. The children were selected for the control group based on the following criteria: (1) did not meet the criteria for a DSM-IV diagnosis of ADHD according to the Korean version of the Diagnostic Interview Schedule for Children Version-IV (DISC-IV) [36,37]; (2) scored below the 90th percentile on the ARS; (3) had a T-score of less than 60 on the Attention Problem subscale of the CBCL; and (4) had an IQ score higher than 71 on the KEDI-WISC.

Subjects were excluded from the study if they had one or more of the following: (1) past and/or current history of any neurological disorder, including a seizure disorder or brain damage; and (2) presence of a comorbid psychosis, Tourette's, bipolar, communication, learning, or pervasive developmental disorder. In the ADHD subjects, ADHD and comorbid disorders were diagnosed using the Korean version of the Kiddle Schedule for Affective Disorders and Schizophrenia-Present and Lifetime Version [38,39].

The study was approved by the institutional review board (IRB) for human subjects at the Seoul National University Hospital and other hospitals. Parents/guardians provided written informed consent, and the children or adolescents provided verbal assent regarding participation in this study.

### Neuropsychological assessments

We used a computerized continuous performance test (CPT) [40] to measure the neuropsychological functions of the ADHD children and controls. The Korean version of the CPT was standardized, and its validity and reliability have been well-established [41]. The four variables recorded included: (1) omission errors (failure to respond to the target), commonly interpreted as a measure of inattention; (2) commission errors (responding inappropriately to the non-target), commonly interpreted as a measure of impulsivity; (3) response times for correct responses to the target, interpreted as a measure of information processing and motor response speed; and (4) the standard deviation of the response times for correct responses to the target (response time variability), which is interpreted as a measure of variability or consistency of attention.

### ADHD children’s emotional symptoms

ADHD children’s emotional symptoms were measured using the Korean versions of the Children’s Depression Inventory (CDI) and the State-Trait Anxiety Inventory for Children (STAIC). The CDI consists of 27 self-report questions along a Likert scale ranging from 0 (not present) to 2 (present and marked), and the total scores on the CDI can range from 0 to 54 [42,43]. The subdomains on the CDI include negative mood, interpersonal problems, negative self-esteem, ineffectiveness, and anhedonia. A total score of 29 is considered the cutoff point for severe depressive symptoms in the Korean version [43]. The STAIC consists of two 20-item scales that measure anxiety level in children [44,45]. One scale is the State-Anxiety Inventory for Children (STAIC-S), which asks the subjects to describe how they feel at the present time and how their anxiety increases in response to situational stress and declines under relaxed conditions. The Trait-Anxiety Scale for Children (STAIC-T) asks the subjects to describe how they generally feel and measures relatively stable individual differences in anxiety proneness. The total score ranges from 20 to 60 for the 20 questions of each scale, and a total score of 49 is considered as the cutoff value for severe anxiety symptoms in the Korean version [45].

### Genotyping

Genomic DNA was extracted from whole blood lymphocytes using a G-DEXTM II Genomic DNA Extraction Kit (Intron, Korea). The detection of a single nucleotide polymorphism was based on an analysis of primer extension products generated from previously amplified genomic DNA using a chip-based matrix-assisted laser desorption/ionization time-of-flight (MALDI-TOF) mass spectrometry platform (Sequenom, California, USA). Primers in the PCR and **homogeneous Mass Extend** (hME) reactions were designed using Assay Designer 3.1 (Sequenom) (5^′^-ACG TTG GAT GCG TCA TCG TGG AAG ACA TAG and 5^′^-ACG TTG GAT GAC GTT CAG GTG ACA GCA TTC for the rs8049651 polymorphism in GRIN2A, 5^′^-ACG TTG GAT GTG GAG ATT TGG TGG AAT GAC and 5^′^-ACG TTG GAT GTG GAG ATT TGG TGG AAT GAC for the rs2284411 polymorphism in GRIN2B, and 5^′^-ACG TTG GAT GTG TGA TAC ACA GCT AAG CAG and 5^′^-ACG TTG GAT GTG AAC CCA AGA CTT CTC ACC for the rs3792452 polymorphism in GRM7).

### Statistical analysis

All data analyses were performed using the Statistical Package for Social Sciences (Version 19.0 for Windows; SPSS, Inc., Chicago, IL, USA). For all data from 202 patients with ADHD and 159 controls, allele frequencies were calculated, and the occurrence of Hardy–Weinberg equilibrium was established by way of goodness-of-fit χ2 tests. Genotype distribution for each polymorphism was in agreement with the expected values of the Hardy-Weinberg equilibrium (p > 0.05). A family-based study was performed to assess genetic association by transmission disequilibrium test (TDT) statistics.

Subjects with ADHD were divided into genotype groups on the basis of their GRIN2A rs8049651, GRIN2B rs2284411, or GRM7 rs37952452 polymorphism. Due to the small number of individuals having two rare alleles, subjects were dichotomized according to whether they possessed the rare A allele (recessive model). Group differences in the continuous clinical variables were examined using an independent two sample t-test. Between-group comparisons with categorical data were assessed using the χ^2^ test or Fisher’s exact test. The significance level was set at p = 0.05 (two-tailed).

## Results

The case–control analysis included 202 ADHD subjects with a mean age of 9.0 +/− 2.5 years and 159 controls with a mean age of 9.0 +/− 2.7 years. The mean ages of the two groups were not significantly different (p = 0.924). However, the gender distribution of the two groups differed significantly, as 174 ADHD subjects (86.1%) and 111 controls (69.8%) were boys (χ2 = 14.67, p < 0.001). Oppositional defiant disorder was the most common (14.4%) comorbid diagnosis, followed by anxiety disorder (6.5%), enuresis (5.5%), and depressive disorder (3.5%) (Table 1). All participants were ethnically Korean. The family-based analysis included 149 trios consisting of an affected subject and his or her biological father and mother.

**Table 1:** Demographic and clinical characteristics of ADHD subjects and controls

	**ADHD (n = 202)**	**Controls (n = 159)**
Age (years), mean ± SD	9.0 ± 2.5	9.0 ± 2.7
Sex (males/females)	174/28	111/48
IQ, mean ± SD	106.1 ± 14.7	114.8 ± 15.3
ADHD subtype, %		
Combined	63.0	
Inattentive	24.5	
Hyperactive-impulsive	6.0	
Not otherwise specified	6.5	
Comorbid disorder, %		
Oppositional defiant disorder	14.4	
Conduct disorder	1.5	
Depressive disorder	3.5	
Anxiety disorder	6.5	
Enuresis	5.5	

Abbreviations: *ADHD* attention-deficit hyperactivity disorder, *IQ* intellectual quotient.

The genotype distribution of the three SNPs did not deviate from expectation based on the Hardy–Weinberg equilibrium (p > 0.05). There were no significant differences in the genotype or allele frequencies of the GRIN2A rs8049651, GRIN2B rs2284411, or GRM7 rs37952452 polymorphisms between the ADHD and control groups (Table 2). For 148 ADHD trios, the TDT analysis also showed no preferential transmission of the GRIN2A rs8049651 or GRIN2B rs2284411 polymorphisms. However, the TDT analysis of the GRM7 rs3792452 polymorphism showed biased transmission of the G allele (χ2 = 4.67, p = 0.031) (Table 3).

**Table 2:** Comparison of the genotype and allele frequencies of GRIN2A, GRIN2B, and GRM7 polymorphisms between ADHD probands and controls

	**ADHD (n = 202)**		**Controls (n = 159)**		**χ2**	**Cohen’s w**	**p**
	**n**	**%**	**n**	**%**			
GRIN2A (rs8049651)							
Genotype					1.73	**0.07**	0.421
GG	179	89.1	137	88.2			
GA	21	10.4	22	13.8			
AA	1	0.5	0	0			
Allele					0.46	**0.03**	0.499
G	379	94.3	299	93.1			
A	23	5.7	19	6.9			
GRIN2B (rs2284411)							
Genotype					1.52	**0.06**	0.469
CC	128	63.7	111	69.8			
CT	66	32.8	43	27.1			
TT	7	3.5	5	3.1			
Allele					1.16	**0.04**	0.281
C	322	80.1	299	83.3			
T	80	19.9	19	16.7			
GRM7 (rs3792452)							
Genotype					1.62	**0.07**	0.444
GG	174	86.6	140	88.1			
GA	25	12.4	19	11.9			
AA	2	1	0	0			
Allele					0.42	**0.02**	0.551
G	373	92.8	299	94			
A	29	7.2	19	6			

**Table 3:** Transmission disequilibrium test results for the GRM7 rs37952452 polymorphism

	**Over-transmitted allele**	**T/NT**	**χ**^**2 **^**(df = 1)**	**Cohen’s w**	**p**
GRIN2A (rs8049651)	G	23/16	1.26	**0.07**	0.262
GRIN2B (rs2284411)	C	58/48	0.94	**0.06**	0.331
GRM7 (rs3792452)	G	28/14	4.67	**0.13**	0.031

Comparisons were assessed using McNemar’s χ2 test; Number of trios = 149.

Table 4 shows the result of the CPT in the ADHD probands according to the genotype at GRM7 rs37952452. A total of 185 ADHD subjects without missing data were included in the analysis. We found no significant differences between excluded (n = 16) and included (n = 185) subjects with regard to demographic and clinical characteristics. There were no significant differences in age, gender, IQ, ARS scores, ADHD subtypes, or comorbidities between ADHD probands with the GG genotype and the other genotypes at GRM7 rs3792452. The subjects who were homozygous for the G allele (GG genotype) in the GRM7 rs37952452 polymorphism had higher mean T-scores for omission errors on the CPT than did those with the GA or AA genotype (t = 3.38, p = 0.001). For the total sample having no missing data (n = 336; 185 ADHD probands and 151 controls), the subjects with the GG genotype in the GRM7 rs37952452 polymorphism also showed higher mean T-scores for omission errors on the CPT than did those with the GA or AA genotype (t = 2.17, p = 0.034). We found no significant differences with regard to commission errors, response time, or response time variability according to the genotypes of GRM7 rs37952452 polymorphism in both ADHD probands and the total sample.

**Table 4:** Comparison of the CPT results according to the genotypes of the GRM7 rs37952452 polymorphism in ADHD probands

	**G/G (n = 161)**	**G/A + AA (n = 24)**	**t**	**Cohen’s d**	**p**
	**Mean (SD)**	**Mean(SD)**			
Omission errors	82.2 (40.9)	62.8 (23.3)	3.38	**0.58**	0.001^*^
Commission errors	85.0 (37.6)	80.8 (32.9)	0.51	**0.77**	0.608
Response time	52.7 (15.6)	49.0 (13.0)	1.11	**1.04**	0.269
Response time variability	87.4 (47.5)	83.1 (37.8)	0.43	**0.10**	0.668

Abbreviations: *CPT* continuous performance test, *ADHD* attention-deficit hyperactivity disorder.

^*^ Significant after Bonferroni correction (0.05/ number of variables of CPT, critical p-value of 0.013).

Table 5 shows the results of the STAIC and CDI in the ADHD probands according to the genotype at GRM7 rs37952452. A total of 169 ADHD subjects without missing data and with no comorbid mood or anxiety disorders were included in this analysis. There were no significant differences in age, gender, IQ, ARS scores, or ADHD subtypes between ADHD probands with the GG genotype and the other genotypes at GRM7 rs3792452. Among the 201 ADHD probands, the frequency of a comorbid anxiety disorder was 11.1% in the ADHD subjects with the GG genotype and 5.7% in those with the GA or AA genotype (X2 = 1.11, p = 0.292). The frequency of comorbid depressive disorder was 7.4% in the ADHD subjects with the GG genotype and 2.3% in those with the GA or AA genotype (X2 = 2.11, p = 0.147). Among the 169 ADHD subjects included in the analysis, the ADHD subjects who were homozygous for the G allele in the GRM7 rs37952452 polymorphism had higher STAIC-T (t = 5.52, p < 0.001) and STAIC-S (t = 2.74, p = 0.007) scores than did those with the GA or AA genotype.

**Table 5:** Comparisons of the CDI, STAI-T, and STAI-S results according to the genotypes of the GRM7 rs37952452 polymorphism in ADHD probands

	**G/G (n = 148)**	**G/A + AA (n = 21)**	**t**	**Cohen’s d**	**p**
	**Mean (SD)**	**Mean(SD)**			
STAI-T	31.8 (10.4)	12.3 (11.1)	5.52	**1.81**	< 0.001
STAI-S	31.4 (10.3)	21.5 (14.2)	2.74	**0.80**	0.007
CDI	14.0 (7.2)	10.9 (7.2)	1.27	**0.43**	0.208

Abbreviations: *ADHD* attention-deficit hyperactivity disorder, *CDI* Children’s Depression Inventory, *STAIC-T* Trait-Anxiety Inventory for Children, *STAIC-S* State-Anxiety Inventory for Children.

No significant associations were found among the results of the CPT, STAIC, or CDI and the GRIN2A rs8049651 or GRIN2B rs2284411 genotypes (data available upon request).

## Discussion

In this study, the case–control and family-based association analyses of the GRIN2A rs8049651 and GRIN2B rs2284411 polymorphisms found no significant association of these two polymorphisms with ADHD in Korean subjects. Previously, Turic et al. [23] reported that the GRIN2A rs8049651 polymorphism was associated with ADHD in a family study, but Adams et al. [21] failed to replicate this positive result. Similarly, Dorval et al. [24] reported that the GRIN2B rs2284411 polymorphism was associated with ADHD symptom severity as well as ADHD diagnosis.

The negative results in our study of these loci that had been previously reported as associated with ADHD could be interpreted in several ways. First, if the GRIN2A rs8049651 and GRIN2B rs2284411 polymorphisms are uncommon disease loci or ones of small effect, then our power to detect a gene would be reduced, and the failure to demonstrate a significant association in our case–control analysis might reflect false negative results stemming from the limited sample size and the resulting lower statistical power. Because susceptibility genes for ADHD are likely to have a small effect size [46], more independent case–control studies with larger sample sizes will be required to ensure sufficient study power to detect small gene effects. Second, there is a possibility that the GRIN2A rs8049651 and GRIN2B rs2284411 polymorphisms might have no involvement in the susceptibility to ADHD, at least in the Korean population. Indeed, ethnic differences may have affected the results. In particular, the allele frequencies for the G allele and for the A allele of the GRIN2A rs8049651 in the ADHD probands of this study were 0.94 and 0.06, respectively, which were very different from those reported in previous studies (0.69, 0.31 for the Turic sample, and 0.72, 0.28 for the Adams sample), suggesting that findings from Western populations may not be generalizable to the Korean population.

With regard to the GRM7 rs37952452 polymorphism, this is the first study to report an association between the GRM7 polymorphism and ADHD using case–control and family-based analyses. The biased transmission of the G allele in the TDT analysis of our ADHD trios suggests a possible role of this allele in the development of ADHD. However, we found no association between the GRM7 rs37952452 polymorphism and ADHD using the case–control approach. As reviewed by Bobb et al. [47], a relatively high percentage (46%) of the 26 genetic studies using the family-based and case–control approaches on the same population and polymorphism found divergent results, indicating that both methods should be used to limit the possibility of type II error. Our positive result in the family-based analysis alongside a negative result in the case–control analysis with regard to the GRM7 rs37952452 polymorphism may be partially explained by the small sample size. The number of patients having a minor allele was only 29 in the ADHD group and 19 in the control group, a disparity which might have negatively impacted statistical precision. Therefore, further case–control association studies with larger sample sizes are necessary. Another potential explanation is that differences in population substructures can affect bias estimates in case–control study designs. However, the Korean population is notably characterized by a relatively high genetic homogeneity. Therefore, stratification bias is unlikely to have affected the result of case–control analyses in our sample. It is also possible that the GRM7 rs37952452 polymorphism might have no involvement in the susceptibility to ADHD, and the positive result in our family-based analysis could be a false positive finding.

In this study, the GRM7 rs37952452 polymorphism was associated with omission errors on the CPT. Numerous studies using neuropsychological tests in individuals with ADHD have found deficits in selective attention [48], and continuous performance tests have been frequently used to evaluate attention deficit in ADHD children [49]. In particular, the omission errors are an indicator of deficits in sustained attention in response to target stimuli. Thus, the results of this study suggest that the GRM7 rs37952452 polymorphism could be more closely linked to the intermediate phenotype of selective attention deficits than to the behavioral phenotype or the disease itself. The intermediate phenotype is used to describe a categorical trait or dimension that usually occurs in an “intermediate” position in the causal chain between genes and disease [50]. Because an intermediate phenotype mediates the relationship between genotype and DSM diagnosis and is more closely connected to genetic functionality, larger magnitudes for genetic effects might be evident and more easily detectable in smaller samples [51]. Furthermore, a relationship between omission error scores and the GRM7 rs37952452 genotype was evident in the total sample (including controls) as well as for ADHD probands, suggesting that this polymorphism might be associated with sub-threshold attention-deficit in the general population.

Although the frequency of a comorbid anxiety disorder did not significantly differ according to the GRM7 rs37952452 genotype, it is notable that the GG genotype of the GRM7 rs37952452 polymorphism was associated with higher state and trait anxiety scores even after excluding the subjects with comorbid anxiety disorders. An association between GRM7 and anxiety has been previously reported. Animal studies, for example, have shown that mGluR7 ablation can cause deficits in fear response [30], and mGluR7 facilitates extinction of aversive memories and controls amygdala plasticity [29]. Elia et al. [52] observed an ADHD proband with a copy number variation in GRM7 gene who also presented with anxiety and suggested that this copy number variation may potentially explain certain comorbidities frequently associated with ADHD. Our results also support the idea that GRM7 rs3795245 polymorphism represents certain phenotypes such as anxious ADHD as well as the ADHD diagnosis itself.

Several limitations of this study should be noted. First, the patient group included all subtypes of ADHD, which might have contributed to clinical heterogeneity. Second, although the genotype distribution was not different according to ADHD subtypes, when we compared the CPT, CDI, and STAI-C results according to the genotypes, the subtypes might have acted as potential confounders. Third, because of the relatively small sample size, the samples of this study would have limited power to detect genes of small effect. Finally, we genotyped only one specific SNP of GRIN2A, GRIN2B, or GRM7, and this may have been insufficient to fully address the association between these glutamate genes and ADHD.

## Conclusions

These results provide preliminary evidence of an association between the GRM7 rs37952452 polymorphism and selective attention deficit and anxiety found within the Korean ADHD population. Further studies, including a replication of these findings with more SNPs in a larger sample, are warranted to evaluate the association of glutamate genes and complex phenotypes of ADHD.

## Abbreviations

ADHD: Attention-deficit hyperactivity disorder; NMDA: N-methyl-D –aspartate; NMDAR: N-methyl-D-aspartate receptor; mGluR: Metabotropic glutamate receptor; GRM7: Metabotropic glutamate receptor mGluR7 gene; DSM-IV-TR: Diagnostic and Statistical Manual of Mental Disorders, 4th edition, text revision; ARS: ADHD Rating Scale-IV; CBCL: Child Behavior Checklist; IQ: Intelligent quotient; DISC-IV: Diagnostic Interview Schedule for Children Version-IV; CPT: Computerized continuous performance test; CDI: Children’s Depression Inventory; STAIC: State-Trait Anxiety Inventory for Children.

## Competing interests

None of the authors have any financial interest in the study, or any other competing interest.

## Authors’ contributions

SWJ, SCC, JWK, HJY, USC, JWS, and HWK participated in data collection. DYC and SWJ analyzed the data. SWJ prepared the first draft of the report. SCC and MSS supervised the statistical analysis. SP and BNK interpreted the results. SP wrote the final report with input from all the authors. All authors read and approved the final manuscript.
